# Relationship Between Symmetrical Peripheral Gangrene Patients and Using Vasopressors in the Intensive Care Unit

**DOI:** 10.7759/cureus.58117

**Published:** 2024-04-12

**Authors:** Turki Alhumam, Aminah A Alhumam, Ibrahim Alhumaidi, Ibrahim Al Rajeh, Yousef Alduhailan

**Affiliations:** 1 College of Medicine, King Faisal University, Al-Hofuf, SAU; 2 Dermatology, King Faisal University, Al-Hofuf, SAU

**Keywords:** septic shock (ss), management of vasopressor-induced acute limb ischemia, vasopressor-induced acute limb ischemia, gangrene in icu, limb ischemia in icu, amputation, disseminated intravascular coagulation (dic), vasopressor complications, digital gangrene, symmetrical peripheral gangrene (spg)

## Abstract

Symmetrical peripheral gangrene (SPG) is a rare yet severe condition characterized by peripheral ischemic lesions without significant vascular occlusion. Its clinical presentation includes peripheral cyanosis, mottling, and symmetrical ischemia of distal limbs, often progressing to gangrene. Recent years have seen a rise in SPG cases, with mortality rates ranging from 40% to 90%. The condition is associated with systemic diseases, such as sepsis, vasculitis, and coagulopathy. DIC frequently complicates SPG, reflecting a disturbed procoagulant-anticoagulant balance and depletion of natural anticoagulants. While vasopressor therapy, particularly high-dose administration, has been implicated in SPG pathogenesis due to sustained vasoconstriction or idiosyncratic responses, recent evidence suggests it may not be the underlying cause. Studies indicate a low incidence of ischemic limb necrosis associated with high-dose vasopressors, with DIC and shock liver potentially explaining limb ischemia instead. The characteristic temporal interval between the onset of shock liver and limb ischemic necrosis suggests a more complex pathophysiology. The role of infectious agents, such as bacteria and viruses, in SPG pathogenesis is under investigation, with both direct vascular invasion and immune-mediated mechanisms proposed. Diagnosis involves ruling out other causes of acral gangrene through clinical examination, laboratory tests, imaging studies, and biopsy. Treatment strategies aim to halt disease progression, eliminate causative factors, and prevent complications. While anticoagulants, vasodilators, and adjunctive therapies like hyperbaric oxygen show promise, the efficacy of interventions varies, emphasizing the need for individualized management. Notably, hemoadsorption has emerged as a promising treatment, demonstrating significant improvement in SPG cases. Amputation remains a last resort option in irreversible cases. Early recognition and multidisciplinary management are crucial for improving outcomes. Further research is needed to better understand SPG's etiology and develop effective treatments through collaborative efforts.

## Introduction and background

Symmetrical peripheral gangrene (SPG) is an uncommon yet catastrophic condition, marked by the emergence of peripheral ischemic lesions in the absence of significant vascular occlusion [1]. As a disseminated intravascular coagulation frequently linked with systemic diseases, such as sepsis, vasculitis, and states of increased blood clotting, SPG presents unique challenges [2].

Clinically, SPG is identified by symptoms including peripheral cyanosis, mottling, and symmetrical ischemia of the distal limbs, often starting in the digits and moving proximally. Additional signs can include skin necrosis, ulceration, and gangrene [2].

Despite its rarity, the frequency of SPG has been on the rise recently, accompanied by alarming mortality rates, which can be as high as 40-90%. [3] The prognosis for SPG tends to be grim, often involving a high likelihood of amputation and death. Recognizing the condition early is pivotal for improving its prognosis, although the effectiveness of early intervention in changing its outcome is still a subject of debate [4]. Understanding this condition is critical, as early detection and intervention are key to preventing complications and improving patient outcomes. Hence, there is a need for heightened awareness and early identification of the underlying causes to prevent life-threatening outcomes and limb loss due to irreversible ischemia and gangrene [5]. Despite advancements in diagnosing and treating SPG, the pathophysiology and etiology of the condition are not fully comprehended.

The objective of our study is to perform an in-depth review of the existing literature on SPG, with a specific focus on the role of inotropes in its pathogenesis. This study is crucial as it augments the current understanding and addresses the gaps in the scarce literature on SPG [1].

## Review

Clinical presentation

The clinical features of SPG can vary, so we will list them as typical and atypical manifestations.

*Typical Manifestations* 

Pain and numbness: Patients often experience severe pain in the affected extremities, which may be disproportionate to the apparent physical findings [1]. Numbness and tingling sensations can also be present [1].

Skin discoloration: The skin in the affected areas may initially appear pale or mottled, indicating poor blood flow. As the condition progresses, it can progress to cyanosis (bluish discoloration) and eventually become dark and gangrenous [2].

Coolness: The affected extremities may feel cold to the touch due to reduced blood flow and impaired circulation [5].

Distal involvement: SPG typically affects the distal parts of the extremities, such as the fingers, toes, and tips of the nose and ears [6]. It often spares the central parts of the limbs [6].

Symmetry: A hallmark feature of SPG is the symmetric involvement of multiple extremities. Both hands and feet are usually affected simultaneously, although the severity may vary [6].

Rapid progression: The development of gangrene can occur rapidly, sometimes within hours or days. It is important to recognize the condition promptly to initiate appropriate management [6].

Absence of major vessel occlusion: Unlike other forms of gangrene, SPG does not result from major arterial occlusion but rather from microvascular dysfunction and thrombosis in the small blood vessels [6].

Atypical Manifestations

We present two atypical cases reported: A case of a 70-year-old female who developed SPG involving both upper extremities (all fingers) and her right foot following severe septic shock necessitating systemic vasopressor therapy [7]. Notably, her left foot remained spared because of a pre-existing chronic external iliac artery occlusion, resulting in a lower concentration of vasopressors in that extremity [7].

Another case shows a 75-year-old Hispanic male patient treated for septic shock after emergency ureteral stent placement surgery. Despite fluid resuscitation, vasopressors, and antibiotics, the patient experienced gangrene in both feet and left hand, with an unusual sparing of the right hand. Subsequently, the patient underwent transmetatarsal amputations of both feet and amputation of three digits of the left hand [8].

These cases underscore the varied and unpredictable presentations of SPG in the context of severe septic shock and the potential influence of pre-existing vascular conditions on disease manifestation.

Etiology

Numerous causal elements have been linked to SPG (Table 1) [9].

**Table 1:** Etiologies of symmetrical peripheral gangrene

Infective	Noninfective
1. Bacterial: *Neisseria meningitidis*, *Streptococcus pneumoniae*, *Staphylococcus aureus*, *Streptococcus pyogenes,* *Klebsiella pneumoniae*, *Salmonella paratyphi*, *Proteus vulgaris*, *Proteus mirabilis*, *Pasteurella multocida*, *Enterococcus faecalis,* *Escherichia coli,* *Mycobacterium*, *Tuberculosis*, *Capnocytophaga*	1. Cardiovascular: myocardial infarction, cardiac failure, hypovolemic shock, hypertension, pulmonary embolism, supraventricular tachycardia
2. Parasitic: *Plasmodium falciparum*	2. Drugs: adrenaline, noradrenaline, dopamine, warfarin, propylthiouracil
3. Viral: viral gastroenteritis, rubeola, varicella zoster, dengue, HIV	3. Malignancy: Hodgkin’s lymphoma, lung adenocarcinoma, adenocarcinoma‑associated thrombotic endocarditis
	4. Connective tissue disorders: systemic lupus erythematosus, polymyalgia rheumatic, antiphospholipid syndrome
	5. Miscellaneous: deficiency of protein C and S, sickle cell disease cryoglobulinemia, suprapubic prostatectomy, emergency neurosurgery

Pathophysiology

There are uncertainties about SPG pathophysiology. The pathophysiology of SPG is intricately tied to three primary factors: DIC, vasopressors, and microbiological elements.

Disseminated Intravascular Coagulation Factor

SPG pathogenesis is characterized by a severely disrupted balance of procoagulant and anticoagulant factors in vulnerable tissue beds [9]. Currently, the most accepted pathogenesis theory is microthrombosis caused by an imbalance of procoagulant and anticoagulant factors [10].

The three main characteristics of SPG are disseminated intravascular coagulation (DIC), protein C and antithrombin deficiency, and shock (hypotension, lactic acidemia, normoblastemia, and multiple organ failure) [10]. Recent research has discovered risk factors for natural anticoagulant depletion, particularly acute ischemic hepatitis, sometimes known as "shock liver," which affects at least 90% of patients with SPG [10]. The development of shock/shock liver and the start of ischemic injury due to peripheral microthrombosis (also known as "limb ischemia with pulses") occur typically two to five days (median three days) [10]. This reflects the amount of time needed for hepatically produced natural anticoagulants to experience significant depletion [10].

Vasopressor Factor

Vasopressors with favorable inotropic effects, like noradrenaline and dopamine, are commonly utilized in septic shock [11]. Administering dopamine at low concentrations, ranging from 2 to 5 mcg/kg/min, leads to the dilation of blood vessels in the coronary, renal, and mesenteric regions.

It increases cardiac contractility at moderate concentrations of 5-20 mcg/kg/min by directly acting on beta-adrenergic receptors and by releasing phenylpropanolamine from tissue storage sites [11]. However, in higher dosages reaching 20-50 mcg/kg/min, dopamine can induce vasoconstriction as a result of stimulating alpha receptors [12]. Alpha-receptor stimulant noradrenaline is frequently used in patients with septic shock; nevertheless, its vasospastic effects can be greater in the digital vascular beds [13]. As a result, peripheral gangrene is not uncommon with large dosages of dopamine or noradrenaline [13]. Furthermore, according to several studies, SPG patients did not have diabetes mellitus or peripheral vascular disease risk factors, and their inotrope therapy was neither extended nor administered at large dosages [13]. Thus, they suggest that the gangrene may have developed as an idiosyncratic response to inotropes [13,14]. The dysregulation of norepinephrine clearance and release is one theory. An imbalance in norepinephrine release and absorption can cause chronic vasoconstriction, which lowers blood flow to the afflicted extremities [14]. This persistent vasoconstriction may cause ischemia and tissue death by depriving the tissues of nutrition and oxygen [14,15]. The stimulation of alpha-adrenergic receptors by norepinephrine is another possible method. Alpha-adrenergic receptors are present on the smooth muscles of blood vessels and are responsible for vasoconstriction [14]. Excessive stimulation of these receptors by norepinephrine can result in persistent constriction of the small arteries and arterioles, further exacerbating ischemia in the affected extremities [14,16].

It is important to note that the role of norepinephrine in SPG is still a subject of ongoing research, and the precise mechanisms involved have yet to be fully elucidated. In addition, other factors, such as endothelial dysfunction, coagulation abnormalities, and microvascular thrombosis, are believed to contribute to the pathogenesis of SPG.

Theoretically, vasopressors may cause limb or digit ischemia; however, a systematic review of ischemic limb necrosis in septic shock and the role of high-dose vasopressor therapy shows that this is not the case and that studies do not rule out shock liver or DIC as a possible explanation for ischemia, nor do they establish a connection between high-dose vasopressor therapy and peripheral ischemia [1]. However, DIC, a serious complication of septic shock that affects around 35% of patients, may be responsible for limb ischemia due to thrombosis occlusion of the microvasculature [1]. While there have been suggestions that high-dose vasopressor medication may cause limb and digit ischemia necrosis, there is no information to confirm this clinical theory or pinpoint the exact prevalence of this illness in this high-risk population [1]. In addition, there exists a distinct temporal sequence in which the initiation of shock liver occurs a median of three days before limb ischemia necrosis occurs. This window of time allows for significant depletion of natural anticoagulants, such as protein C and antithrombin [3,10,17]. If vasopressors were the true cause of limb ischemia, it should occur as an immediate consequence, instead of three days later, on average [1].

*Microbiological Factor* 

The role of infectious agents in the pathogenesis of SPG has been a topic of interest among researchers for years. Several studies have suggested that bacterial and viral infections can lead to the development of SPG through either direct or indirect mechanisms.

Direct mechanisms of infection-related SPG pathogenesis may involve the direct invasion of blood vessels by microorganisms, leading to endothelial dysfunction and damage. Certain gram-negative bacteria, such as *Neisseria meningitidis*, have been implicated in the development of SPG through this mechanism. These bacteria produce endotoxins that activate the coagulation cascade and lead to the formation of microvascular thrombi, causing ischemia and tissue necrosis [4]. The most frequently implicated microorganisms are pneumococcus, streptococcus, and staphylococcus [4].

Indirect mechanisms through Shwartzman reaction of infectious SPG pathogenesis involve the production of inflammatory cytokines and other mediators by the immune system in response to infection. These mediators can cause endothelial dysfunction, activate the coagulation cascade, and lead to microvascular thrombi and peripheral ischemia [2,10,18]. Similarly, parasitic infections, such as *Plasmodium falciparum,* have been associated with the development of SPG through the production of inflammatory cytokines and activation of the coagulation cascade [19,20].

While the exact mechanisms by which infectious agents contribute to SPG pathogenesis are still not fully understood, it is clear that infections can play a significant role in the development of this severe and life-threatening condition. Identification and early treatment of the underlying infectious agent are crucial for the management of SPG in infected patients.

Differential diagnosis

To confirm an SPG diagnosis, all possible causes of acral gangrene should be ruled out. Thromboangitis obliterans, atherosclerosis, thromboembolic gangrene, secondary Raynaud’s phenomenon, diabetes, neuropathy, chemical or toxic agents, calciphylaxis, and vasculitis gangrene are potential differential diagnoses. Sparing the main arteries, suggestive history and natural course, and absence of signs of vasculitis in histology should differentiate SPG from these diseases [9].

Investigation

Diagnosing SPG can be a challenge as its symptoms often mimic those of other conditions, such as ischemic muscle necrosis, arterial embolism, or thrombosis. Therefore, it is crucial to perform a comprehensive evaluation to rule out other differential diagnoses and to confirm the diagnosis of SPG.

This can be done through a combination of clinical examination, laboratory tests, and imaging studies [9].

Clinical examination plays a crucial role in the diagnosis of SPG. The hallmark clinical presentation of SPG is symmetrical and painful peripheral gangrene, usually affecting the distal extremities, such as the hands, feet, and toes. The skin over the affected area is often dusky and cold to the touch and may have bullae formation. Nail changes, such as loss of nails, can also be seen [9].

Laboratory tests can also provide important information in the diagnosis of SPG. Complete blood cell (CBC) count, erythrocyte sedimentation rate (ESR), and coagulation profile and screening for DIC are some of the initial tests that should be performed [1].

ESR can suggest an underlying systemic disease that may be causing SPG. Serological tests, such as antiphospholipid antibody (APLA), lupus anticoagulant (LA), or ANA, can also be performed to rule out underlying autoimmune conditions [9].

Imaging studies play a crucial role in the diagnosis and management of SPG. Doppler ultrasound is the most commonly used non-invasive imaging modality and can be used to evaluate the arterial and venous circulation of the affected limb. Angiography, on the other hand, is a more invasive but more specific test that can provide a clear image of the arterial anatomy and occlusions. Magnetic resonance angiography (MRA) and computed tomography angiography (CTA) may also be performed to evaluate other causes [9].

The most accurate method of diagnosing SPG pathology is biopsy [6]. The majority of SPG specimens exhibit microthrombi in the superficial and deep vascular plexus capillary lumen, along with fibrin deposition and mild red blood cell extravasation [9,21]. The biopsy specimen may contain subepidermal cell poor bulla, but there are no signs of inflammatory infiltrates or vasculitis in the vascular walls [9].

A study including the skin biopsies of three patients reveals that the petechial appearance of early lesions is influenced by edematous endothelial cells, capillary dilatation, and red cell extravasation. These lesions eventually merge into the region of ischemia necrosis with concomitant bullae [6,22].

This will first affect the dermal and subdermal tissues. When significant peripheral necrosis occurs, it might affect the underlying tissues and bone [6,21].

It is important to search for microangiopathic changes, such as schistocytes, in a peripheral blood smear since their presence might indicate the possibility of DIC. After multiorgan failure, postmortem examinations of the patients' organs reveal microthrombi in the kidneys (cortical necrosis), lungs, liver, spleen, adrenal glands, heart, brain, pancreas, and gastrointestinal system [6,21].

Treatment

There are not many evidence-based recommendations for treating SPG. However, the widely accepted objective in treating this condition is to stop the disease's progression, eliminate causative factors, prevent additional infections, address hypovolemia, and excise dead tissue. A multidisciplinary management strategy is essential, involving a team of healthcare professionals including internists or critical care experts, dermatologists, hematologists, and general or orthopedic surgeons. Admission to a critical care unit is necessary. Managing shock resulting from blood loss and sepsis typically involves substantial fluid replenishment [4].

Treatment of Underlying Conditions

The management of SPG involves the treatment of the underlying conditions. Infections, connective tissue disorders, malignancies, and other systemic conditions should be treated as appropriate.

Low-dose heparin therapy is reported to be effective in halting the progression from pre-gangrenous changes to frank gangrene in SPG. Sharma et al. (2004) suggested the use of heparin (300-500 iu/hour) for this purpose [4]. Furthermore, the theoretical involvement of heparin-based anticoagulation in SPG treatment is supported by its association with a disturbed procoagulant-anticoagulant balance, as highlighted by Patankar (2020) [23].

A comprehensive approach to SPG treatment often includes anticoagulants alongside antibiotics and supportive care, as demonstrated by Mala and Ahmed (2022) [24]. This is in line with the inclusion of anticoagulation therapy in treatment regimens that also involve corticosteroids and anti-malaria therapy, as noted by Shahin (2015) [25].

The role of natural anticoagulants and heparin in SPG treatment is also under consideration. Foead et al. (2018) discussed the theoretical involvement of heparin-based anticoagulation and substitution of natural anticoagulants in SPG management [26]. Alternative anticoagulant therapies, such as anti-platelet medication and pentoxifylline, have also been explored, with cases showing resolution in SPG, as reported by Liao et al. (2015) [18].

However, the effectiveness of anticoagulant and antiplatelet agents has been questioned, with some studies suggesting that the initial pathophysiology of SPG might be more vasoconstrictive than thrombotic [27]. In addition, there have been instances where anticoagulopathic treatment failed to prevent amputation, as observed by Schuh et al. (2002) [10,28], who also argued that a causal role for anticoagulant therapy in SPG is unproven and unlikely [10]. Meanwhile, anticoagulation, especially with heparin, is a common element in SPG treatment, and its efficacy can vary. Individualized treatment plans, considering the underlying disease and patient's condition, are recommended for SPG management.

Vasodilators, such as prostaglandins, particularly prostacyclin (PGI2), have vasodilatory and antiplatelet effects, which may theoretically contribute to improving microcirculatory flow and reducing ischemic injury in the extremities affected by SPG. Prostaglandins, through their vasodilatory properties, may help in improving peripheral perfusion and tissue oxygenation, potentially mitigating the progression of ischemic lesions in the extremities [14]. A case reported in 2018 showed successful improvement of the lesion using prostaglandins and low-molecular-weight heparin [14]. In addition, innovative methods such as botulinum toxin injections offer promise in managing SPG by promoting vasodilation and alleviating cutaneous ischemia ​[29].

There have been positive reports in the literature on the use of epoprostenol and recombinant tissue plasminogen activator together [4,26,30]. However, there is still a need for more research and clinical trials to establish their efficacy and optimal use in this condition [4,26,30]. Sympathectomy, by interrupting sympathetic nerve control, can effectively reverse this vasoconstriction and restore blood flow. However, its effectiveness is limited if the ischemia is caused by thrombosis due to conditions, like DIC or vascular damage from immune complexes, where the issue is vessel occlusion rather than mere vasoconstriction [31].

Hyperbaric Oxygen Therapy (HBOT)

HBOT involves the use of oxygen at high pressure to improve oxygen delivery to ischemic tissues. Some studies have described good outcomes with the use of HBOT in the management of SPG [32,33]. The study shows that HBOT improved the symptoms and perfusion of the affected limbs in patients with SPG [3,33].

Hemoadsorption (HA) is a method of extracorporeal blood purification through a cartridge, where solutes are removed by direct binding to the sorbent material. Hemoadsorption is used to control cytokine levels in different settings. According to data from an international registry, whole-blood cytokine adsorber therapy reduced IL-6 levels in 68% of patients with sepsis. A study reported that early and aggressive management of septic shock, including the use of hemoadsorption, can help to stop the progression of SPG and to prevent amputation from being required. Reducing cytokine levels can halt the progression of gangrene and improve recovery chances in SPG patients [34].

The last therapeutic option is amputation considered a last-resort treatment when the extent of tissue damage is irreversible and limb salvage is not feasible. The decision to amputate is usually made based on the extent of the necrosis, the patient's overall health, and the likelihood of recovery with other treatments [35]. Amputation of the gangrenous area was rarely urgent, and many cases were auto amputation [3]. Several authors came to the conclusion that early amputation should be avoided since the initial estimate of tissue destruction may not be accurate and viable tissue may be sacrificed during the procedure [3].

Amputation can range from the removal of fingers and toes to more significant limb amputations, depending on the severity and spread of the gangrene [35]. Post-amputation, patients often require rehabilitation and may need prosthetics to aid in mobility and improve quality of life [35].

## Conclusions

Symmetrical peripheral gangrene is a severe condition with a poor prognosis, often resulting in death or amputation. DIC is frequently observed in these patients and is likely the culmination of the microvascular damage underlying the syndrome's characteristic symptoms. Since SPG is uncommon, no controlled trials have been conducted on any particular therapy; hence, the emphasis is still on managing DIC and determining and treating its underlying causes. Early detection and awareness of SPG are crucial for promptly initiating comprehensive, team-based patient care. Further research is needed to deepen our understanding of the condition's origins and develop effective treatment approaches, ideally through collaborative efforts across multiple medical centers.
